# An Organic/Inorganic Hybrid Membrane as a Solid “Turn-On” Fluorescent Chemosensor for Coenzyme A (CoA), Cysteine (Cys), and Glutathione (GSH) in Aqueous Media

**DOI:** 10.3390/s120302969

**Published:** 2012-03-02

**Authors:** Saúl Vallejos, Pedro Estévez, Saturnino Ibeas, Félix C. García, Felipe Serna, José M. García

**Affiliations:** Departamento de Química, Facultad de Ciencias, Universidad de Burgos, Plaza de Misael Bañuelos s/n, E-09001 Burgos, Spain; E-Mails: svallejos@ubu.es (S.V.); paeb14@gmail.com (P.E.); sibeas@ubu.es (S.I.); fegarcia@ubu.es (F.C.G.); fserna@ubu.es (F.S.)

**Keywords:** sensory materials, chemosensor, fluorogenic sensor, biomolecules, sensing biomolecules

## Abstract

The preparation of a fluorogenic sensory material for the detection of biomolecules is described. Strategic functionalisation and copolymerisation of a water insoluble organic sensory molecule with hydrophilic comonomers yielded a crosslinked, water-swellable, easy-to-manipulate solid system for water “dip-in” fluorogenic coenzyme A, cysteine, and glutathione detection by means of host-guest interactions. The sensory material was a membrane with gel-like behaviour, which exhibits a change in fluorescence behaviour upon swelling with a water solution of the target molecules. The membrane follows a “turn-on” pattern, which permits the titration of the abovementioned biomolecules. In this way, the water insoluble sensing motif can be exploited in aqueous media. The sensory motif within the membrane is a chemically anchored piperazinedione-derivative with a weakly bound Hg(II). The response is caused by the displacement of the cation from the membrane due to a stronger complexation with the biomolecules, thus releasing the fluorescent sensory moieties within the membrane.

## 1. Introduction

The development of sensing molecules for the detection of chemicals is a topic of current interest [1–6]. The recognition of target molecules based on the variation of a macroscopic property of a sensing molecule associated with the specific interactions of the target with the receptor motifs of the sensor can be used to prepare sensory solutions for the easy, cheap and rapid quantification of chemicals by means of a widely used analytical technique (e.g., UV/Vis and/or spectrofluorometry). Moreover, if the receptor and the transducing motifs are chemically bound to a polymer network structure, then the organic material can be described as a solid system, which can potentially be used as a solid kit for the “dip-in” detection of analytes [3].

For medical, biomedical and environmental reasons, biological molecules are among the most important target analytes [7]. Biomolecules containing a thiol group, such as coenzyme A (CoA), l-cysteine (Cys), and glutathione (GSH), play important roles in biological processes including acyl group carrier capability or oxidation/reduction facility (*i.e.*, intramolecular reduction-oxidation metabolic cycles), which occur in hundreds of biochemical reactions [8–12]. The determination of the biomolecule concentration has been undertaken with various methodologies. One of the most interesting is the sensing methodology based on the host-guest supramolecular approach. The supramolecular approach has recently been applied to the determination of Cys [13–27], CoA [8–10], and GSH to a lesser extent [28].

**Scheme 1.**
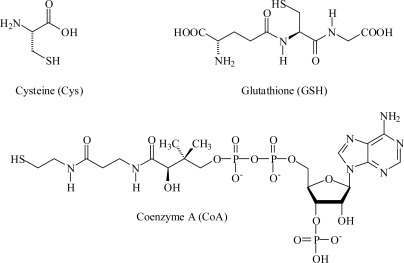
Structure of Cys, GSH, and CoA.

Herein, we describe a sensory organic/inorganic hybrid membrane for the fluorogenic detection of three important biomolecules: CoA, Cys, and GSH (the chemical structures are shown in Scheme 1). The membrane was a dense film consisting of a hydrophilic acrylic network that contained a small amount of piperazinedione-derivative/Hg(II) moieties as the sensory motif toward the biomolecules mentioned above. The piperazinedione-derivative was chemically anchored to the copolymer backbone. The water-swelled membrane responded to the presence of the targets in an aqueous environment at physiological pH with an increase in the fluorescence intensity (*i.e.*, a fluorescence “turn-on” pattern), which permitted the titration of the biomolecules.

## 2. Experimental Section

### 2.1. Materials

The following commercially available materials and solvents were used as received, unless otherwise indicated: mercury(II) acetate (Sigma Aldrich, 98%), glycine (Sigma Aldrich, 99%), methacryloyl chloride (Fluka, 97%), ethylene glycol dimethacrylate (Aldrich, 98%), ethylene glycol (Fluka), acetic anhydride (Sigma Aldrich, puriss.), potassium *t*-butoxide (Sigma Aldrich, 99.99%), 4-(dimethylamino)benzaldehyde (Sigma Aldrich, 98%), triethylamine (Fluka, 99.5%), lithium chloride (Sigma Aldrich, 99%), 4-nitrobenzaldehyde (Sigma Aldrich, 99%), sodium sulphide nonahydrate (Sigma Aldrich, 98%), dioxane (Probus, 99%), 1-vinyl-2-pyrrolidone (Sigma Aldrich, 99%), N-methyl-2-pyrrolidone (Sigma Aldrich, 99.5%), diethyl ether (VWR, 99.99%), DMSO (Merck, 99%), acetone (Aldrich, 99%), ethanol (Aldrich, 99%), methanol (VWR, for HPLC), DMF (Aldrich, 99%), coenzyme A trilithium salt (Calbiochem, 99.9%), l-glutathione reduced (Alfa Aesar, 97%), and l-cysteine hydrochloride monohydrate (VWR). Azo-bis-isobutyronitrile (AIBN, Fluka, 98%) was recrystallised twice from methanol.

### 2.2. Measurements

^1^H and ^13^C-NMR spectra were recorded in deuterated dimethyl sulphoxide (DMSO-*d*_6_) as the solvent using a Varian Inova 400 spectrometer operating at 399.92 and 100.57 MHz, respectively. Infrared spectra (FTIR) were recorded with a Nicolet Impact spectrometer or with a JASCO FT/IT-4100 fitted with a PIKE TECH “Miracle” ATR. Thermogravimetric analysis (TGA) data were recorded using 5 mg of sample under a nitrogen or oxygen atmosphere on a TA Instrument Q50 TGA analyzer at a scan rate of 10 °C min^−1^. UV-Vis spectra were recorded using a Varian Cary3-Bio UV-Vis spectrophotometer. The fluorescence spectra were recorded using a Varian Cary Eclipse fluorometer. Millipore-Q water was used to prepare the solutions. To determine the tensile properties of the membranes, strips (5 mm in width, 30 mm in length, and 30–45 μm thick) were cut from the polymer films and measured using a Hounsfield H10KM Universal Testing Dynamometer at 20 °C. Mechanical clamps held the sample, and an extension rate of 5 mm min^−1^ was applied using a gauge length of 10 mm. At least six samples were tested for each polymer, and the data were averaged.

### 2.3. Intermediates and Monomer Synthesis

**Scheme 2.**
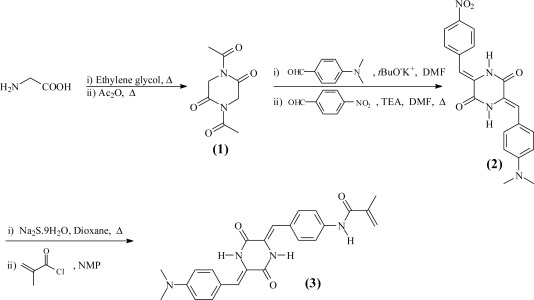
Synthesis of the monomer *N*-(4-((1*Z*)-((*Z*)-5-(4-(dimethylamino)benzylidene)-3,6-dioxopiperazin-2-ylidene)methyl)phenyl)methacrylamide.

The overall synthetic steps for the monomer are shown in Scheme 2.

*Synthesis of 1,4-diacetylpiperazine-2,5-dione* (**1**). Glycine (a total of 100 g, 1.33 mol) was dissolved in ethylene glycol (500 mL) in a 1,000 mL flask fitted with a mechanical stirrer. The mixture was stirred at 170 °C for 3 h, and the solution was cooled at 5 °C for 20 h. The precipitate, piperazine-2,5-dione, was collected by filtration and washed with methanol (500 mL). Then, the solid was dissolved in boiling water, and the solution was cooled overnight. The white product was filtered off and washed with methanol. Yield: 30%. M.p.: 330 °C. ^1^H-NMR δ_H_ (399.9 MHz, DMSO-*d*_6_, Me_4_Si): 4.64 (2H, s, NH); 3.85 (4H, s, CH_2_). ^13^C-NMR, δ_C_ (100.6 MHz, DMSO-*d*_6_, Me_4_Si): 168.46, 43.83. EI-LRMS m/z: 114 (M^+•^, 100), 86 (8), 72 (2), 58 (5), 56 (7). FTIR [wavenumbers (cm^−1^)]: ν_N-H_: broadband (3,250, 2,750); ν_C=O_: 1,696.

Piperazine-2,5-dione (18.7 g, 0.164 mmol) and acetic anhydride (85 mL) were added to a 250 mL flask equipped with a reflux condenser. The mixture was stirred at reflux for 7 h. The solvent was removed by distillation. The product **1** was washed with diethyl ether and collected by filtration. Yield: 90%. M.p.: 96 °C. ^1^H-NMR δ_H_ (399.9 MHz, DMSO-*d*_6_, Me_4_Si): 4.64 (6H, s, CH_3_); 3.84 (4H, s, CH_2_). ^13^C-NMR, δ_C_ (100.6 MHz, DMSO-*d*_6_, Me_4_Si): 173.61, 168.17, 43.32, 26.12. EI-LRMS m/z: 198 (M^+•^, 30), 156 (41), 114 (47), 86 (3), 71 (30), 43 (12). FTIR [wavenumbers (cm^−1^)]: ν_N-C=O_: broadband (3,452, 3,365); ν_C=O_: 1,718.

*Synthesis of (3Z,6Z)-3-(4-(dimethylamino)benzylidene)-6-(4-nitrobenzylidene) piperazine-2,5-dione* (**2**). A flask equipped with a reflux condenser was charged with 1,4-diacetylpiperazine-2,5-dione (**1**, 4.94 g, 25 mmol) and 4-(dimethylamino)benzaldehyde (3.72 g, 25 mmol) which were dissolved in DMF (70 mL). Potassium *t*-butoxide (2.8 g, 25 mmol) was added, and the mixture was stirred at room temperature for 12 h. The product, (*Z*)-3-(4-(dimethylamino)benzylidene)-1-acetylpiperazine-2,5-dione, was precipitated in water and collected by filtration. Finally, the solid was washed with water and methanol. Yield: 50%. M.p.: 210 °C. ^1^H-NMR δ_H_ (399.9 MHz, DMSO-*d*_6_, Me_4_Si): 10.19 (1H, s, NH); 7.53 (2H, d, *J* 8.7, ArH); 6.95 (1H, s, CH); 6.79 (2H, d, *J* 9.0, ArH); 4.39 (2H, s, CH_2_); 3.02 (6H, s, CH_3_); 2.51 (3H, s, CH_3_). ^13^C-NMR, δ_C_ (100.6 MHz, DMSO-*d*_6_, Me_4_Si): 172.31, 165.00, 163.19, 151.28, 132.07, 122.99, 122.45, 120.86, 112.39, 46.08, 26.96. EI-LRMS m/z: 287 (M^+•^, 90), 245 (100), 160 (52), 115 (3), 78 (11), 62 (14). FTIR [wavenumbers (cm^−1^)]: ν_N-H_: broadband (3,661, 3,310); ν_C=O_: 1,696, 1,597 and 1,521.

(*Z*)-3-(4-(dimethylamino)benzylidene)-1-acetylpiperazine-2,5-dione (2.6 g, 9 mmol) and 4-nitro-benzaldehyde (1.36 g, 9 mmol) were dissolved in DMF (135 mL) in a flask equipped with a reflux condenser. Triethylamine (0.9 g, 9 mmol) was added, and the mixture was stirred at 130 °C for 12 h. A solid was collected by filtration and washed with methanol. Finally, the solid was washed with acetone at its reflux temperature in a flask equipped with a reflux condenser. Yield: 50%. M.p.: not observed (the compound was amorphous; however, an exothermic crystallisation peak was observed at 353 °C). ^1^H-NMR δ_H_ (399.9 MHz, DMSO-*d*_6_, Me_4_Si): 10.43 (1H, s, NH); 10.09 (1H, s, NH); 8.20 (2H, d, *J* 6.42, ArH); 7.75 (2H, d, *J* 7.35, ArH); 7.43 (2H, d, *J* 6.42, ArH); 6.74 (4H, t, *J* 5.93, ArH); 2.95 (6H, s, CH_3_). EI-LRMS m/z: 378 (M^+•^, 100), 332 (3), 287 (1), 215 (1), 159 (37), 117 (5), 89 (4), 77 (1). FTIR [wavenumbers (cm^−1^)]: ν_N-H_: broadband (3,628, 3,331), ν_N-H_: 3,211; ν_C=O_: 1,674 and 1,626; ν_NO_: 1,578 (asymmetric) and 1,339 (symmetric).

*Synthesis of N-(4-((1Z)-((Z)-5-(4-(dimethylamino)benzylidene)-3,6-dioxopiperazin-2-ylidene)methyl) phenyl)methacrylamide* (**3**). In a 250 mL flask fitted with a reflux condenser, compound **2** (1.8 g, 4.75 mmol) was dissolved in dioxane (100 mL). Sodium sulphide nonahydrate (3.43 g, 14.25 mmol) was added to the solution, and the mixture was stirred at 80 °C for 24 h. The solution was filtered, and water (400 mL) was added. The resultant precipitate, (3*Z*,6*Z*)-3-(4-(dimethylamino) benzylidene)-6-(4-aminobenzylidene) piperazine-2,5-dione, was filtered off and washed twice with methanol. Yield: 72%. M.p.: 307 °C. ^1^H-NMR δ_H_ (399.9 MHz, DMSO-*d*_6_, Me_4_Si): 9.81 (2H, s, NH_2_); 7.47 (2H, d, *J* 9.03, ArH); 7.32 (2H, d, *J* 9.03, ArH); 6.78 (2H, d, *J* 10.08, ArH); 6.69-6.61 (4H, m, ArH); 5.63 (2H, s, NH); 3.00 (6H, s, CH_3_). ^13^C-NMR, δ_C_ (100.6 MHz, DMSO-*d*_6_, Me_4_Si): 159.26, 159.12, 150.66, 150.08, 131.66, 131.43, 123.50, 122.82, 121.29, 120.99, 117.10, 116.41, 114.36, 112.60. EI-LRMS m/z: 348 (M^+•^, 100), 334 (2), 306 (1), 218 (1), 161 (17), 159 (22), 133 (24), 131 (10). FTIR [wavenumbers (cm^−1^)]: ν_N-H_: 3,432, 3,340 and 3,229; ν_C=O_: 1,672 and 1,598.

In a 25 mL flask fitted with a reflux condenser and under N_2_ atmosphere, (3*Z*,6*Z*)-3-(4-(dimethylamino)benzylidene)-6-(4-aminobenzylidene) piperazine-2,5-dione (1.2 g, 3.45 mmol) was dissolved in NMP (7 mL). Methacryloyl chloride (0.47 g, 4.5 mmol) was added to the solution, and the mixture was stirred at room temperature for 4 h. An orange solid (monomer **3**) was collected by filtration and purified from the crude residue by washing with hot acetone using a Soxhlet apparatus. Yield: 80%. M.p.: 330 °C. ^1^H-NMR δ_H_ (399.9 MHz, DMSO-*d*_6_, Me_4_Si): 10.08 (2H, s, NH); 9.97 (1H, s, NH); 7.79 (2H, d, *J* 8.7, ArH); 7.56 (2H, d, *J* 8.7, ArH); 7.48 (2H, d, *J* 8.7, ArH); 6.77 (4H, m, ArH); 5.86 (1H, s, CH_2_); 5.58 (1H, s, CH_2_); 3.01 (6H, s, CH_3_); 1.99 (3H, s, CH_3_). ^13^C-NMR, δ_C_ (100.6 MHz, DMSO-*d*_6_, Me_4_Si): 167.77, 159.55, 159.02, 151.09, 141.26, 139.89, 131.89, 130.74, 129.22, 126.63, 123.43, 121.44, 121.14, 120.91, 117.64, 115.12, 112.91, 19.80. EI-LRMS m/z: 416 (M^+•^, 100), 376 (16), 347 (4), 159 (62), 131 (9), 117 (5), 77 (3). FTIR [wavenumbers (cm^−1^)]: ν_N-H_: broadband (3,709, 3,100); ν_C=O_: 1,676, 1,624 and 1,595.

### 2.4. Membrane Preparation

**Figure 1.**
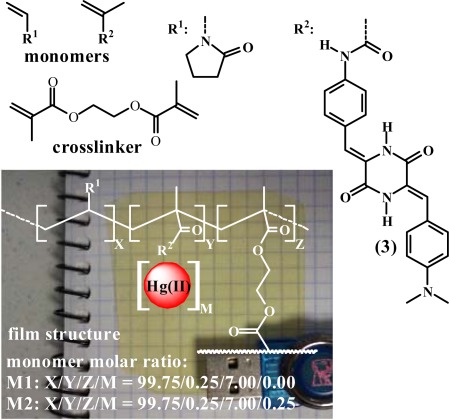
Chemical structures of the monomers and the copolymer. The copolymer is shown over a digital picture of the sensory film.

Membrane **M1** was prepared by the radical polymerisation of a mixture of 1-vinyl-2-pyrrolidone and (**3**) with a molar ratio of 99.75:0.25. Ethylene glycol dimethacrylate was used as the cross-linking agent (7% mol percentage regarding the overall comonomer molar content), and AIBN (1 wt%) was used as a thermal radical initiator. Membrane **M2** was prepared following the same procedure described for the preparation of **M1**; however, 0.25% molar content of mercury(II) acetate was added (the same concentration of (**3**)), which resulted in a hybrid organic-inorganic material. The thermal polymerisation was performed in 100 μm thick silanised glass moulds in an oxygen-free atmosphere at 65 °C for 5 h. The structure and the physical appearance are depicted in Figure 1.

## 3. Results and Discussion

### 3.1. Material Characterisation

Mechanical and thermal resistance are key parameters to determine the suitability of an organic material for technological applications. From a mechanical point of view, **M1**, a dense membrane, showed good performance. The Young’s modulus was 490 MPa and the elongation at break was 160% at room temperature with a relative humidity of 65%. The hydrophilic membranes were dried at 103 °C for 20 minutes, which resulted in an increase in the Young′s modulus to 1.1 GPa and a decrease in the elongation at break to 12%. The membrane recovered the initial values upon exposure to the ambient atmosphere. The hydrophilic character of the material resulted in a water uptake of 150% upon immersing the membrane in pure water. A comparison between the FTIR spectra of dry **M1** and **M1** stored in air overnight (under the abovementioned conditions) was performed. The band corresponding to the amide I of the hydrophilic vinylpyrrolidone moieties exhibited a band shift toward lower energy of 18 cm^−1^ (1,668 to 1,648 cm^−1^), while the shoulder at 1,727 cm^−1^ that corresponds to the hydrophobic ester residue of the crosslinker remained unchanged. These observations probably indicate that hydrophilic and hydrophobic microdomains were present in the water-swelled membrane after immersing the membrane in aqueous media for sensing purposes.

**Figure 2.**
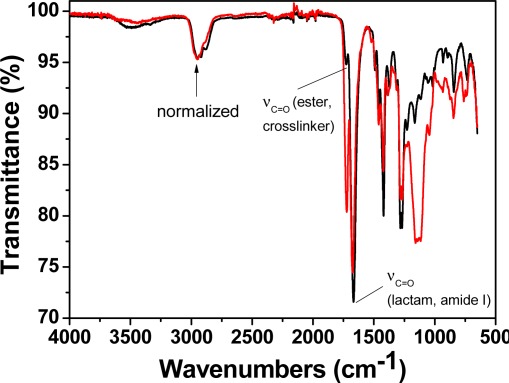
FTIR spectra of the dried membranes **M1** (black line) and **M2** (red line).

The polymerisation of the comonomers without and with low molar content mercury (II) acetate (0.25%) (**M1** and **M2**, respectively) resulted in materials with fairly different FTIR spectra (Figure 2). Comparing the spectra of dry samples of **M1** and **M2** resulted in the observation of an intense band that developed at 1,722 cm^−1^ for **M2**, with a concomitant shift of the amide I band to higher energies, from 1,668 to 1,675 cm^−1^, probably due to the acetate group.

**Figure 3.**
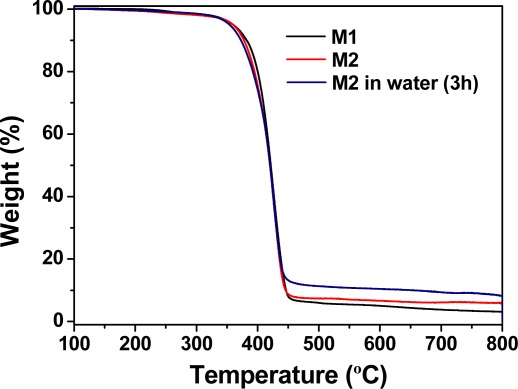
TGA curves of membranes **M1** and **M2**. The degradation pattern of **M2** after a cycle of soaking in pure water for 3 h with subsequent drying at rt is also included.

The thermal resistance of the membranes was evaluated using TGA. The decomposition temperatures that resulted in 5% and 10% weight loss under a nitrogen atmosphere (*T*_5_ and *T*_10_, respectively) were approximately 360 and 385 °C, which indicates the material had reasonably good thermal stability. **M1** and **M2** had a first weight loss at 200 °C, which was attributed to the non-reticulated chain ends [29]. The TGA curves of the membranes are shown in Figure 3. The residue remaining after reaching 800 °C was negligible for **M1** and approximately 8% for **M2**, which confirms the influence of the mercury content in the thermal behaviour. The mercury was first oxidised to HgO, which indicates the hybrid nature of the membrane. The immersion of membrane **M2** in water resulted in an insignificant loss of bound Hg(II), as determined by comparing the amount of residue that remained at 800 °C under a nitrogen atmosphere for two samples of **M2** that were soaked in pure water for 3 and 24 h and subsequently dried. Both samples resulted in a residue of 8%. Nevertheless, the analysis of the role of the Hg(II) by TGA is cumbersome, because of the behaviour of the mercury salts upon heating. Initially, mercury oxides formed, and then, metallic mercury was formed with concomitant sublimation [30]. Changing the atmosphere from nitrogen to air yielded the complete loss of mass at 800 °C for **M1** and **M2**, which gave rise to a zero char yield.

### 3.2. The Membranes as Sensory Materials

**Figure 4.**
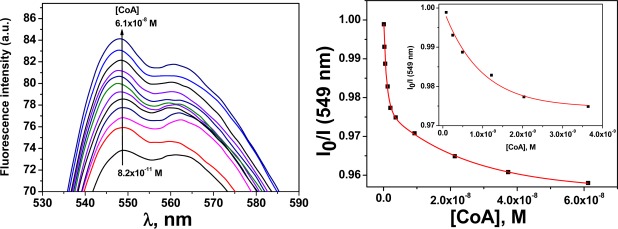
Selected fluorescence spectra (left) and titration curve (right) of **M2** upon adding increasing quantities of CoA in water at physiological pH (pH = 7.4, TRIS) at an excitation wavelength of 400 nm. The inset for the figure on the right is an expansion of the lower concentrations of the titration curve.

The membrane **M1** behaves as a sensory material for the fluorogenic detection of Hg(II) in aqueous media. Upon the addition of Hg(II), the fluorescence of the membrane at 548 nm was quenched, which demonstrated that the membrane had “turn-off” fluorescence behaviour in the presence of the cation. This observation was attributed to the interaction of the Hg(II) with the N-terminus of the sensory motif (**3**) within the membrane at a 1:1 stoichiometry [31]. The integral preparation of a membrane containing equal molar quantities of (**3**) and Hg(II) (*i.e.*, **M2**), led to a material with a partially quenched fluorescence. Moreover, fluorescence recovery was observed for **M2** upon adding different biomolecules, e.g., CoA, Cys and GSH (see Figure 4). The stronger interaction of these biomolecules with Hg(II) led to a fluorescent chemosensor with fluorescence “turn-on” behaviour, based on the displacement approach [1,2]. A titration curve of the biomolecules was obtained by plotting the fluorescence maxima *versus* the biomolecule concentration. An illustrative example is shown for CoA in Figure 4. The limit of detection (LOD) was approximately 2 × 10^−10^ M.

### 3.3. Copolymer Network/Hg(II) Interaction

**Figure 5.**
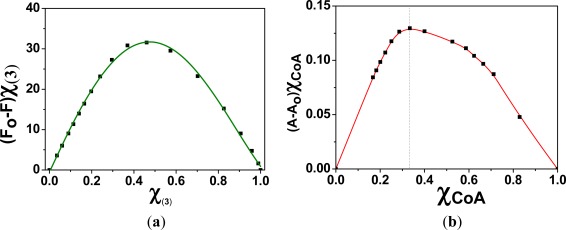
Job’s plots corresponding to the following interaction studies: (**a**) (**3**) with Hg(II), obtained from fluorescence spectroscopy (591 nm) data corresponding to the titration curve of (**3**) with mercury cations in DMSO/water (90/10, v/v) at pH = 9.7 (TRIS); and (**b**) CoA with Hg(II), from UV/Vis spectroscopy (300 nm) data corresponding to the titration of CoA with mercury cations in DMSO/water (90/10, v/v) at pH = 7.4 (TRIS).

Prior to the preparation of the membrane **M2**, the interaction of the monomer containing the sensing motif (**3**) with Hg(II) was studied in solution. The stoichiometry of the (**3**):Hg(II) complexes in a DMSO/water solution (90:10) was mathematically determined by analysing the fluorescence quenching process, the intensity maxima variations *versus* the Hg(II) concentration, and the corresponding Job’s plots. The Job’s plot showed a maximum that appeared at a mole fraction for (**3**) (χ_(**3**)_) of 0.5, which clearly indicated the formation of complexes with a 1:1 stoichiometry, as shown in Table 1 and Figure 5.

The strength of the interaction of (**3**) with Hg(II) in buffered DMSO/water (90/10) solution (pH = 7.4) in terms of the stability constant, *K*_1_, corresponding to the host-guest complexes, was analysed by fluorescence spectroscopy. The determination of the 1:1 stoichiometry of the (**3**):Hg(II) complexes allowed the following equilibrium to be stated:

(1) $$L+M\overset{K_{1}}{⇌}\mathrm{ML}$$

which can also be written as the following:

(2) $$K_{1}=\frac{\left[\mathrm{ML}\right]}{\left[L]\;[M\right]}$$

where [*L*], [*M*], and [*ML*] is the equilibrium concentration of (**3**), Hg(II), and the (**3**):Hg(II) complex, respectively. From the mass balance and fluorescence data, the following equations can be deduced:

(3) $$C_{L}=[L]+[\mathrm{ML}]$$

(4) $$C_{M}=[M]+[\mathrm{ML}]$$

(5) $$I_{F}=f_{L}[L]+f_{\mathrm{ML}}\;[\mathrm{ML}]$$

where *C*_L_, *C*_M_, *I_F_*, *f*_L_, and *f*_ML_ are the total concentration of (**3**), total concentration of Hg(II), fluorescence intensity, and the fluorescence proportional factors of (**3**) and (**3**):Hg(II), respectively.

By solving the equilibrium concentration of (**3**) ([*L*]) from Equation (3) and substituting the value in Equation (5), the following expression was obtained:

(6) $$I_{F}=f_{L}\;C_{L}+[\mathrm{ML}]\;(f_{\mathrm{ML}}-f_{L})$$

where *f*_L_*C*_L_ is the fluorescence intensity of (**3**) upon absence of complex. Thus, given that Δ*F* = *I_F_* – *f*_L_*C*_L_ and Δf = *f*_*M*L_ – *f*_L_, Equation (6) can be transformed into the following expression:

(7) $$[\mathrm{ML}]=\frac{\Delta F}{\Delta f}$$

From Equation (1), and considering Equation (3) to (7), Equation (8) can be deduced:

(8) $$K_{1}=\frac{\left[\mathrm{ML}\right]}{\left[L]\;[M\right]}=\frac{\frac{\Delta F}{\Delta f}}{\left(C_{L}-\frac{\Delta F}{\Delta f}\right)\;\left(C_{M}-\frac{\Delta F}{\Delta f}\right)}$$

Equation (9) can be deduced from Equation (8), which is the following:

(9) $$\frac{C_{L}\;C_{M}}{\Delta I_{F}}+\frac{\Delta I_{F}}{\Delta f^{2}}=\frac{1}{K_{1}\;\Delta f}+\frac{1}{\Delta f}\left(C_{L}+C_{M}\right)$$

Thus, the experimental data could be fitted using an iterative process starting with a tentative value of Δ*f*, representing the left term of Equation (9) *versus* the sum of the concentration of (**3**) and Hg(II), *C*_L_+*C*_M_. The value of Δ*f* was calculated from the slope of the straight line obtained, and the process was repeated until the tentative and the calculated Δ*f* values were approximately equal. The fitting of the experimental data with Equation (8) yielded the following results: *K*_1_ = 110,000 M^−1^ ± 10,000, Δ*f* = −2,000,000 ± 4,000, and *R^2^* = 0.9999.

### 3.4. Interaction of CoA, Cys, and GSH with Hg(II)

**Figure 6.**
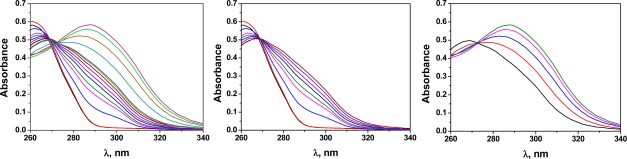
UV/Vis spectra corresponding to the interaction studies of CoA with Hg(II) (left). The figures in the middle and on the right are spectra corresponding to the complexation of the first and second Hg(II) molecules to each sensory molecule (**3**), respectively. Two isosbestic points can be clearly observed.

The interaction of CoA with Hg(II) was studied by UV/Vis spectroscopy, because CoA is not fluorescent. As depicted in the Job’s plot shown in Figure 5, the results indicate that the complex stoichiometry was 1:2 [CoA:Hg(II)]. The stoichiometry could be clearly observed in the UV/Vis spectra, where two equilibria could be estimated (Figure 6).

The equilibrium constants were determined using the following equations:

(10) $$\begin{array}{ccc} M+L\;\;\overset{K_{1}}{⇌} & \text{ML}, & K_{1}=\frac{\left[\mathrm{ML}\right]}{\left[L]\;[M\right]} \end{array}$$

(11) $$\begin{array}{ccc} \text{ML}+M\;\;\overset{K_{2}}{⇌} & M_{2}L, & K_{2}=\frac{{\left[\mathrm{ML}\right]}_{2}}{\left[L]\;[\mathrm{ML}\right]} \end{array}$$

where [*M*], [*L*], [*ML*] and, [*M_2_L*] are the equilibrium concentration of Hg(II), CoA, the Hg(II):CoA complex, and Hg(II)_2_:CoA complex, respectively.

The absorbance was measured at a wavelength where L did not absorb, and an absorbance balance was accounted for to obtain the following expression:

(12) $$A=\varepsilon_{L}[L]+\varepsilon_{\mathrm{ML}}\;[\mathrm{ML}]+\varepsilon_{M_{2}L}\;[M_{2}L]$$

and considering Equations (10) and (11):

(13) $$\left[\mathrm{ML}]=K_{1}\;[M]\;[L\right]$$

(14) $$\left[M_{2}L]=K_{1}\;K_{2}{\left[M\right]}^{2}\;[L\right]$$

Then, combining Equations (12) to (14), Equation (15) was deduced:

(15) $$A=\varepsilon_{L}[L]+\varepsilon_{\mathrm{ML}}\;K_{1}\;[M]\;[L]+\varepsilon_{M_{2}L}\;K_{1}\;K_{2}{\left[M\right]}^{2}\;[L]$$

Using a mass balance, where C_L_ is the total concentration of the ligand (CoA) and C_M_ is the total metal concentration (Hg), results in the following equations:

(16) $$C_{L}=[L]+[\mathrm{ML}]+[M_{2}L]$$

(17) $$C_{M}=[M]+[\mathrm{ML}]+2[M_{2}L]$$

Combining Equations (15)–(17) results in Equation (18):

(18) $$A=\frac{C_{L}}{1+K_{1}\;[M]+K_{1}\;K_{2}\;{\left[M\right]}^{2}}\;\left(\varepsilon_{L}+\varepsilon_{\mathrm{ML}}\;K_{1}\;[M]+\varepsilon_{M_{2}L}\;K_{1}\;K_{2}\;{\left[M\right]}^{2}\right)$$

The values of ε_L_ and ε_M2L_ were estimated as ε_L_ = A_o_/C_L_ and ε_M2L_ = A_final_/C_L_. The concentration of [M] was not known; however, C_M_ was used as an approximation. With these considerations, a nonlinear least-squares regression was conducted on the data, as depicted in Table 1 (ε_ML_ = 16,000 ± 700).

Unfortunately, the study of the interaction of Cys, and GSH with Hg(II) could not be performed because the overlap of the absorbance of the biological molecules and the mercury salt in the UV/Vis spectra. Nevertheless, a similar behaviour as the CoA:Hg(II) complex would be expected.

### 3.5. Interaction of the Membrane **M2** with CoA, Cys and GSH

**Figure 7.**
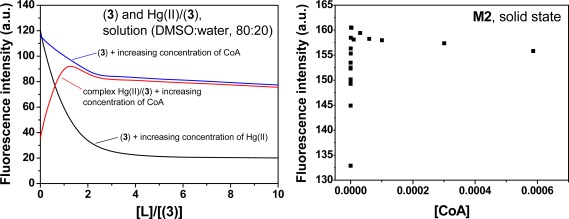
Cross interaction CoA, Hg(II) and (**3**) in DMSO:H_2_O (80:20) solution (left), and interaction of the hybrid membrane **M2** with CoA in water (pH = 7.4, TRIS) (right).

Following the previously described procedure for the study of the interaction of **3** with Hg(II), the complex stoichiometry of (**3**):X (X = CoA, Cys and GSH) and the stability constants were determined following the fluorescence quenching of **3** in solution. The results are shown in Table 1 and have an equimolar complex stoichiometry in all cases. These results show that the biomolecules also interact with the sensory motif **3** within the membrane causing the fluorescence quenching; however, the interaction was significantly weaker than the interaction between Hg(II) and **3**. The following complex behaviour of the sensory material **M2** was observed upon adding the biomolecules to the measurement media: (a) an initial recovery of the fluorescence was observed until a maxima was reached, which corresponded to the displacement of the Hg(II) bound to the sensory motif in **M2** due to the interaction of Hg(II) and the biomolecule; and (b) the quenching of the fluorescence upon increasing the concentration of the biomolecule passed the maxima due to the interaction between the sensory moieties of **M2** and the biomolecule. This phenomenon was also observed studying the system in solution, as depicted in Figure 7. As pointed out it Section 3.1 and shown in Figure 4, a titration curve to measure CoA, Cys and GSH at nanomolar concentrations could be drawn. The upper concentration detection limit depended on the amount of the sensing monomer within the membrane bound to Hg(II), which could easily be modified by varying the monomer feed ratio in the membrane synthesis. The ratio of the monomer to Hg(II) was preferably 1:1, so the concentration window that the sensory material could measure was tunable. The interaction strengths between the three target molecules and the sensory motif were similar, as was estimated in solution by the stability constants (see Table 1), which permits a similar sensing behaviour of the hybrid film **M2** in relation to the fluorescence “turn-on” pattern for detecting the biomolecules.

## 4. Conclusions

This work describes the preparation of a fluorogenic sensory material for the detection of biomolecules such as CoA, Cys, and GSH, in water under physiological conditions. The material is a hybrid membrane with gel-like behaviour, which upon interaction with the target molecules showed a turn “on” fluorescence pattern that permitted the molecules to be titrated. The receptor sensory motif within the membrane was a chemically anchored piperazinedione-derivative with bound Hg(II). The response arose from the displacement of the Hg(II) from the membrane due to a stronger complexation of Hg(II) with the biomolecules, thus releasing the fluorescent sensory moieties. The material recovered the fluorescence behaviour characteristic of the chemical structure of the receptors.

## Figures and Tables

**Table 1.** Stability constants corresponding to the complex (3):X [X = Hg(II), CoA, Cys and GSH], and CoA:Hg(II).

**Complex**	**Complex stoichiometry**	***K*_1_ (M^−1^)**	***K*_2_ (M^−1^)**
**(3)**:Hg(II)	1:1	110,000 ± 10,000	–
**(3)**:CoA	1:1	22,000 ± 2,000	–
**(3)**:Cys	1:1	20,000 ± 8,000	–
**(3)**:GSH	1:1	20,000 ± 7,000	–
CoA:Hg(II)	1:2	8,400 ± 900	6,000 ± 3,000

## References

[1] García J.M., García F.C., Serna F., de la Peña J.L. (2011). Fluorogenic and chromogenic polymer chemosensors. Polym. Rev.

[2] Martínez-Máñez R., Sancenón F. (2003). Fluorogenic and chromogenic chemosensors and reagents for anions. Chem. Rev.

[3] Kim H.N., Guo Z., Zhu W., Yoon J., Tian H. (2011). Recent progress on polymer-based fluorescent and colorimetric chemosensors. Chem. Soc. Rev.

[4] Jeong Y., Yoon J. (2011). Recent progress on fluorescent chemosensors for metal ions. Inorg. Chim. Acta.

[5] Gale P.A. (2011). Anion receptor chemistry. Chem. Commun.

[6] Descalzo A.B., Máñez Martínez-Máñez R., Sancenón Hoffmann K., Rurack K. (2006). The supramolecular chemistry of organic–inorganic hybrid materials. Angew. Chem. Int. Ed.

[7] Zhou Y., Xu Z., Yoon J. (2011). Fluorescent and colorimetric chemosensors for detection of nucleotides, FAD and NADH: Highlighted research during 2004–2010. Chem. Soc. Rev.

[8] Li J., Ge X., Jiang C. (2007). Spectrofluorometric determination of trace amounts of coenzyme A using terbium ion-ciprofloxacin complex probe in the presence of periodic acid. Anal. Bioanal. Chem.

[9] Peng Q., Ge X., Jiang C. (2007). A new spectrofluorometric probe for the determination of trace amounts of CoA in injection, human serum and pig livers. Anal. Sci.

[10] Yu F., Xi C., Li Z., Cui M., Chen F., Gao Y., Chen L. (2009). Enoxacin-Tb^3+^ complex as an environmentally friendly fluorescence probe for Coenzyme A and its applications. Anal. Lett.

[11] Raoof J.B., Ojani R., Kolbadinezhad M. (2009). Voltammetric sensor for glutathione determination based on ferrocene-modified carbon paste electrode. J. Solid State Electrochem.

[12] Wood Z.A., Schröder E., Harris J.R., Poole L.B. (2003). Structure, mechanism and regulation of peroxiredoxins. Trends Biochem. Sci.

[13] Xu H., Gao S., Liu Q., Pan D., Wang L., Ren S., Ding M., Chen J., Liu G. (2011). A highly sensitive and selective competition assay for the detection of cysteine using mercury-specific DNA, Hg^2+^ and SYBR green I. Sensors.

[14] Zhang D., Zhang M., Liu Z., Yu M., Li F., Yi T., Huang C. (2006). Highly selective colorimetric sensor for cysteine and homocysteine based on azo derivatives. Tetrahedron Lett.

[15] Wei X., Qi L., Tan J., Liu R., Wang F. (2010). A colorimetric sensor for determination of cysteine by carboxymethyl cellulose-functionalized gold nanoparticles. Anal. Chim. Acta.

[16] Zhang M., Yu M., Li F., Zhu M., Li M., Gao Y., Li L., Liu Z., Zhang J., Zhang D., Yi T., Huang C. (2007). A Highly selective fluorescence turn-on sensor for cysteine/homocysteine and its application in bioimaging. J. Am. Chem. Soc.

[17] Kim T.K., Lee D.N., Kim H.J. (2008). Highly selective fluorescent sensor for homocysteine and cysteine. Tetrahedron Lett.

[18] Duan L., Xu Y., Qian X., Wang F., Liu J., Cheng T. (2008). Highly selective fluorescent chemosensor with red shift for cysteine in buffer solution and its bioimage: Symmetrical naphthalimide aldehyde. Tetrahedron Lett.

[19] Wang Y., Xiao J., Wang S., Yang B., Ba X. (2010). Tunable fluorescent sensing of cysteine and homocysteine by intramolecular charge transfer. Supramol. Chem.

[20] Yang X.F., Liu P., Wang L., Zhao M. (2008). A chemosensing ensemble for the detection of cysteine based on the inner filter effect using a rhodamine B spirolactam. J. Fluoresc.

[21] Jung H.S., Han J.H., Pradhan T., Kim S., Lee S.W., Sessler J.L., Kim T.W., Kang C., Kim J.S. (2012). A cysteine-selective fluorescent probe for the cellular detection of cysteine. Biomaterials.

[22] Zhou X.-B., Chan W.H., Lee A.W.M., Yeung C.C. (2011). Ratiometric fluorescent probe for enantioselective detection of D-cysteine in aqueous solution. Beilstein J. Organic Chem.

[23] Lim S.Y., Kim H.J. (2011). Ratiometric detection of cysteine by a ferrocenyl Michael acceptor. Tetrahedron Lett.

[24] Yao Z., Bai H., Li C., Shi G. (2011). Colorimetric and fluorescent dual probe based on a polythiophene derivative for the detection of cysteine and homocysteine. Chem. Commun.

[25] Ravindran A., Mani V., Chandrasekaran N., Mukherjee A. (2011). Selective colorimetric sensing of cysteine in aqueous solutions using silver nanoparticles in the presence of Cr^3+^. Talanta.

[26] Xu Z., Yoon J., Spring D.R. (2010). Fluorescent chemosensors for Zn^2+^. Chem. Soc. Rev.

[27] Chen X., Ko S.K., Kim M.J., Shin I., Yoon J. (2010). A thiol-specific fluorescent probe and its application for bioimaging. Chem. Commun.

[28] Zeng X., Zhang X., Zhu B., Jia H., Yang W., Li Y., Xue J. (2011). A colorimetric and ratiometric fluorescent probe for quantitative detection of GSH at physiologically relevant levels. Sens. Actuat. B Chem.

[29] Jablonski A.E., Lang A.J., Vyazovkin S. (2008). Isoconversional kinetics of degradation of polyvinylpyrrolidone used as a matrix for ammonium nitrate stabilization. Thermochim. Acta.

[30] Tariq S.A., Hill J.O. (1981). Thermal analysis of mercury(I) sulfate and mercury(II) sulfate. J. Thermal Anal.

[31] Vallejos S., Estévez P., Ibeas S., Muñoz A., García F.C., Serna F., García J.M. (2011). A selective and highly sensitive fluorescent probe of Hg^2+^ in organic and aqueous media: the role of a polymer network in extending the sensing phenomena to water environments. Sens. Actuat. B Chem.

